# A Multi-Channel Asynchronous Neurostimulator With Artifact Suppression for Neural Code-Based Stimulations

**DOI:** 10.3389/fnins.2019.01011

**Published:** 2019-09-27

**Authors:** Sahar Elyahoodayan, Wenxuan Jiang, Huijing Xu, Dong Song

**Affiliations:** ^1^Department of Biomedical Engineering, Center for Neural Engineering, University of Southern California, Los Angeles, CA, United States; ^2^Neuroscience Graduate Program, University of Southern California, Los Angeles, CA, United States

**Keywords:** brain, deep brain stimulation, hippocampal memory prosthesis, intracortical stimulation, multiplexing, artifact suppression

## Abstract

A novel neurostimulator for generating neural code-based, precise, asynchronous electrical stimulation pulses is designed, fabricated, and characterized. Through multiplexing, this system can deliver constant current biphasic pulses, with arbitrary temporal patterns, and pulse parameters to 32 electrodes using one pulse generator. The design also features a stimulus artifact suppression (SAS) technique that can be integrated with commercial amplifiers. Using an array of CMOS switches, electrodes are disconnected from recording amplifiers during stimulation, while the input of the recording system is shorted to ground through another CMOS switch to suppress ringing in the recording system. The timing of the switches used to block and suppress the stimulus artifact are crucial and are determined by the electrochemical properties of the electrode. This system allows stimulation and recording from the same electrodes to monitor local field potentials with short latencies from the region of stimulation for achieving feedback control of neural stimulation. In this way, timing between each pulse is controlled by inputs from an external source and stimulus magnitude is controlled by feed-back from neural response from the stimulated tissue. The system was implemented with low-power and compact packaged microchips to constitute an effective, cost-efficient, and miniaturized neurostimulator. The device has been first evaluated in phantom preparations and then tested in hippocampi of behaving rats. Benchtop results demonstrate the capability of the stimulator to generate arbitrary spatio-temporal pattern of stimulation pulses dictated by random number generators (RNGs) to control magnitude and timing between each individual biphasic pulse. *In vivo* results show that evoked potentials elicited by the neurostimulator can be recorded ∼2 ms after the termination of stimulus pulses from the same electrodes where stimulation pulses are delivered, whereas commercial amplifiers without such an artifact suppression typically result in tens to hundreds of milliseconds recovery period. This neurostimulator design is desirable in a variety of neural interface applications, particularly hippocampal memory prosthesis aiming to restore cognitive functions by reinstating neural code transmissions in the brain.

## Introduction

Neural interface technology has made much progress in recent years aiming to advance basic neuroscience research and provide therapies to patients with neurological damages or diseases. Researchers have been using neural interface as a tool to record and manipulate neural circuits to study neural correlates of sensory, motor, and cognitive functions (Brecht et al., 2004; Nimmagada and Weiland, 2018; Salas et al., 2018). In the clinic, deep brain stimulation (DBS) has provided treatments to various neurological disorders such as epilepsy (Lee et al., 2015), depression (Schläpfer and Kayser, 2014), Parkinson’s disease (Voges et al., 2007), memory loss (Hamani et al., 2008), and Tourette’s syndrome (Ackermans et al., 2006). In neural prosthesis applications, neural interface has been used to convert sensory input signals to neural stimulations as in cochlea prostheses (Holden et al., 2013) and retinal prostheses (Weiland et al., 2011), or decode motor cortical output signals into movements as in motor prostheses (Velliste et al., 2008).

Hippocampal memory prosthesis is a novel form of neural prosthesis that aims to restore cognitive functions lost in injures or diseases due to destruction of neurons and their connections in a specific region of the brain (Berger et al., 2011). It relies on a computational model that mimics the non-linear dynamical multi-input, multi-output (MIMO) properties of the neural circuit to be replaced (Song et al., 2007, 2018). The MIMO model enables the prosthesis to stimulate a downstream brain region with appropriate output spatiotemporal patterns of neural codes predicted from input spatiotemporal patterns of neural activities recorded from an upstream brain region (Song et al., 2009; Berger et al., 2010). By reinstating the neural code processing and transmission, the damaged brain region is bypassed, and the cognitive function is thus restored (Berger et al., 2011; Hampson et al., 2012, 2013, 2018). To implement a hippocampal memory prosthesis, it is essential to be able to deliver temporally, and spatially distributed neural code-based stimulation patterns to the brain tissue with multiple electrodes.

**FIGURE 1:**
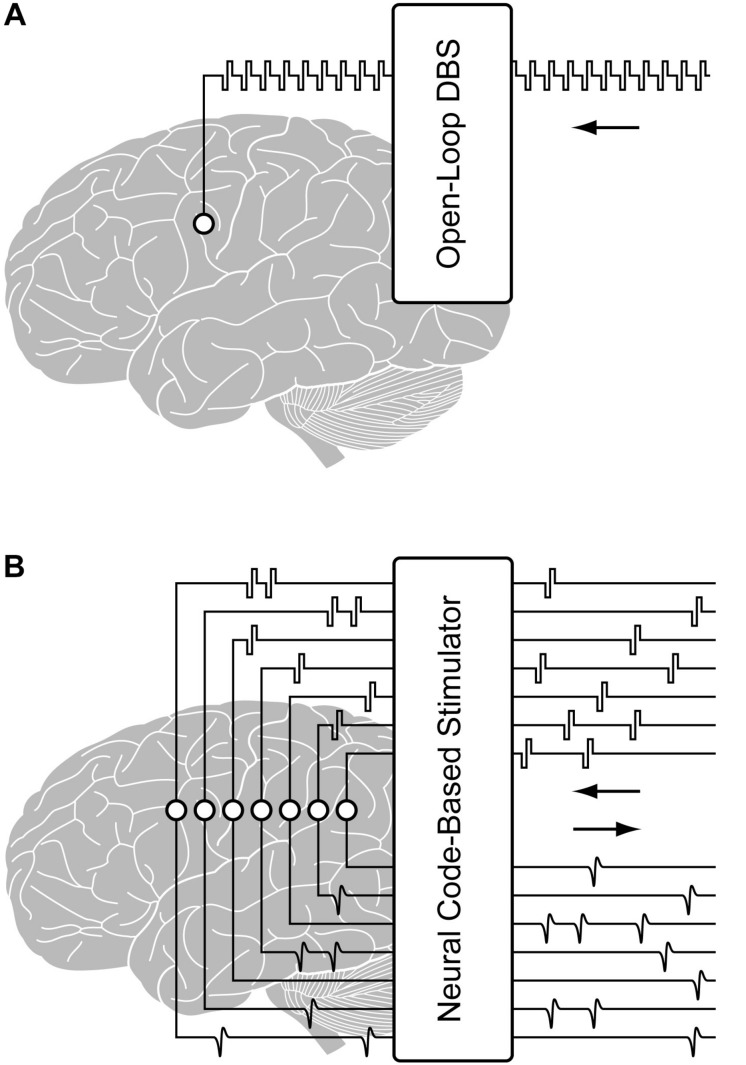
Illustration of **(A)** a conventional open-loop DBS and **(B)** a configurable multi-channel neurostimulator with stimulus artifact suppression (SAS) for implementing closed-loop processing from the region of stimulation.

Existing neural interface technologies for brain implantation, as in DBS, utilize fixed interval trains of pulses, with a single or small number of stimulation electrodes. However, to enable neural code-based stimulation, as required by the hippocampal memory prosthesis, a system for real-time precise delivery of large-scale spatiotemporal patterns of electrical pulses must be designed and implemented. In addition, the ability to stimulate and record from the same electrode is essential for mimicking the neural code (Figure 1). This capability would maximize the number of electrodes for recording or stimulation, provide feedback from stimulated tissue for validating stimulation effects and optimizing stimulation parameters, and enable building single neuron-level MIMO model. These features are also desirable in applications such as the closed-loop DBS for treatment of neurological disorders. For example, multi-channel DBS can provide more focal and effective modulations to the brain compared with single-channel DBS (Vesper et al., 2002; Swan et al., 2018). DBS parameters such as the pulse width, waveform, frequency, amplitude, and duration can be more effectively tuned based on feedback from neural activities (Little et al., 2013; Priori et al., 2013; Salam et al., 2016). Thus, a configurable multi-channel neurostimulator with feedback recording capability may potentially enable safer, more precise and efficient DBS treatments, as well.

The main challenge of generating large-scale stimulation pulses is hardware efficiency. Power and area are crucial parameters of an implantable and apparent solution of separate pulse generators for each electrode is inefficient for a hippocampal memory prosthesis. This is because the natural rate of neural activity is slow, so each pulse generator would need to spend a major amount of time in quiescent mode, which stills consumes power. Whereas, time multiplexing a single pulse generator into multiple channels enables combining several current sources into a single high rate pulse generator. Furthermore, neural spiking activity is sparse in nature and simultaneous pulse generation from multiple electrodes would not result in sparse neural code. On the other hand, asynchronous low magnitude stimulation pulsing has the potential of generating sparse neural code. Accordingly, a highly configurable and asynchronous neurostimulator would enable assessment of whether sparse low amplitude pulses would generate sparse neural code.

The main challenge of recording in conjunction with stimulation is due to the prolonged saturation of the recording amplifier caused by stimulus artifacts, which masks neural activities of interest. Stimulus artifact may last for tens of milliseconds or even hundreds of milliseconds depending on the amplifier, electrode property, tissue property, and stimulus parameters (Rolston et al., 2009). Minimizing such recording contaminants is vital for better recording evoked neural activities and controlling neural interface devices.

In this paper, we present a highly configurable asynchronous multi-channel neurostimulator that can be driven by a MIMO model-based computational unit to continuously generate neural code-like spatiotemporal patterns of stimulation pulses with adjustable pulse parameters. Stimulation pulses may be generated in real-time driven by an external source (i.e., the output of the MIMO model) and feedback from neural response to stimulation from the stimulated tissue. Each stimulation channel is equipped with a switching mechanism designed to reduce recovery period from the artifact from tens to hundreds of milliseconds to ∼2 ms. The system has been designed, fabricated and characterized first in phantom preparations and then the hippocampi of behaving rats. *In vivo* recording demonstrates recovery of early onset compound potentials. Recording of spike activity is also possible with this system based on results from phantom recordings. However, since the same electrode is used for stimulation and recording, the surface area of the electrode must be large for a charge storage capacity that allows delivering enough charge to the tissue to evoke a response. Thus, here the geometric area of the electrode limits recording of spikes.

## Design

The principle elements of the design include a stimulation pattern generator, a multiplexer, a micro-processor-based controller, and a set of serially controlled CMOS switches for stimulus artifact suppression (SAS).

### Neural Code-Based Stimulator Circuit

**FIGURE 2:**
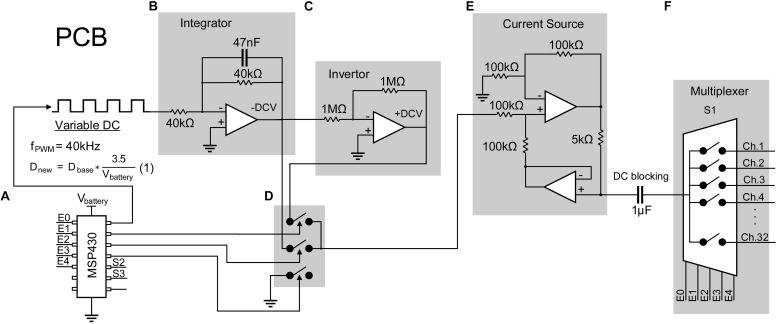
Design of a 32-channel configurable constant current biphasic neurostimulator. It includes **(A)** a microcontroller (MSP430) which generates a PWM at a 40 kHz frequency and an adjustable duty cycle to configure the stimulus amplitude. The duty cycle is also automatically adjusted as the battery supply voltage drops, governed by equation 1, avoiding the need for a regulator. PWM then goes through **(B)** an integrator to generate a constant -DCV, **(C)** an inverter to generate a + DCV, **(D)** a set of analog switches dictating polarity and duration of the pulse controlled by the microcontroller, and **(E)** a voltage to current converter and a DC blocking capacitor to generate safe single channel constant current biphasic pulses. **(F)** Lastly, a multiplexer is used to expand the design to 32 channels.

The stimulator consists of a configurable constant current biphasic and monopolar waveform generator and a pattern generator independently specifying spatiotemporal timings and magnitudes of pulses across 32 stimulating electrodes. First, a single-channel configurable current source capable of providing charge-balanced biphasic pulses is designed and tested as follows. A low-power microcontroller (MSP430G2553, Texas Instrument) is programmed to generate a 40 kHz pulse width modulator (PWM). An op-amp integrator with a cut-off frequency of 4 kHz is designed and used to average the PWM to output a negative DC voltage, which is then inverted to output a positive DC voltage using an inverting amplifier. Three other signals from the microcontroller are generated to drive analog switches (TS5A22362) dictating polarity and duration of each pulse or the inter-pulse intervals. An op-amp-based current source is designed to convert the output voltage biphasic pulses to constant current biphasic pulses with a 1 μA resolution. The output of the current pulse is then fed into a multiplexer to expand a single channel to 32 channels (Figure 2). A DC blocking capacitor is placed at the input of the multiplexer to block input off-set. Since the application of the design requires sparse and low pulse rate, charge build-up is not expected as the electrode is shorted to ground after each stimulation pulse.

This circuit is powered by two 3.7 V coin batteries connected in series to obtain ±3.7 V. The absolute maximum supply rating is determined by the microcontroller, which is +4.1 V. Other chips have a maximum voltage rating of ±5 V. To minimize hardware design for future miniaturization of the PCB, voltage regulators are eliminated since all chips can operate at a minimum voltage of ±3 V. To ensure the output DC voltage from the PWM stays constant even with voltage supply drop, the microcontroller is programmed to sample the supply voltage and adjust the PWM duty cycle according to equation 1:

(1) Dn⁢e⁢w=Dbase*⁢(3.7Vbattery)

where D_new_ is the adjusted duty cycle; D_base_ is the target duty cycle for when the battery voltage is 3.7 V, and V_battery_ is the voltage of the battery at a time point.

### Stimulus Artifact Suppression Technique

Neural recording systems consist of small-signal (typically 10 μV–10 mV) voltage amplifiers with adjustable gain and bandpass filters. Beyond power supply voltage after amplification, the amplifier cannot produce amplification of the input signal. Not only does a large signal cause signal distortion and loss of neural signal, the excess power transfer may damage the amplifier over time. A stimulation pulse applied to an electrode is a large signal and when used in conjunction with a neural recording system, it produces high amplitude artifacts with long recovery period caused by amplifier saturation and filter ringing.

**FIGURE 3:**
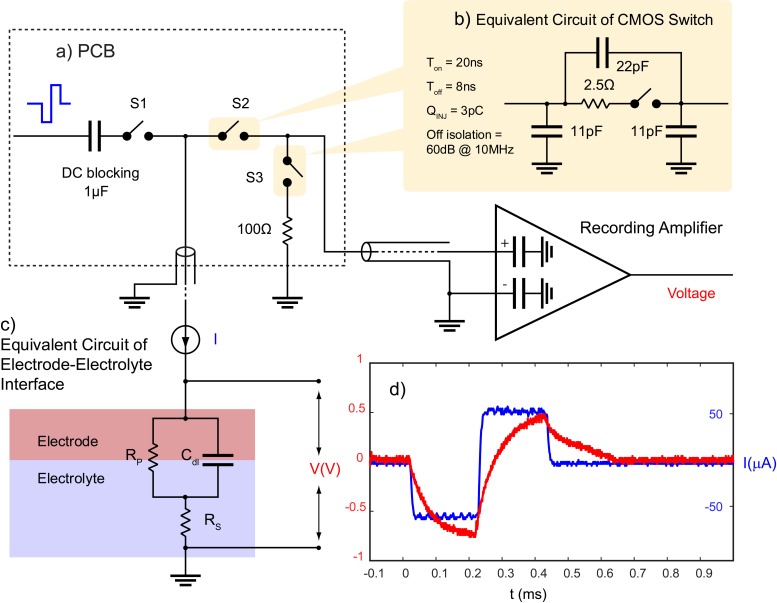
Stimulation artifact suppression set-up. **(a)** Stimulation is synchronized with a set of serially controlled CMOS switches (S2) to block out the stimulus from the recording amplifier during stimulation while connecting the amplifier input to a 100 Ω resistor via S3 to prevent ringing caused in the recording system due to any extra charge coupled across S2. Charge coupling across S2 is due to **(b)** parasitic components of analog switches. The PCB is connected to the recording amplifier and **(c)** the electrode using a coaxial cable. The timing of the switches in **(a)** is determined by **(c)** the electrochemical properties of the electrode, and **(d)** the voltage transient across a microelectrode (red) in response to a biphasic cathodic first current pulse (blue). **(c)** R_S_ represents the electrolyte resistance, *R*_P_ represents faradaic reaction, and *C*_dl_ represents capacitive reactions at the interface.

To prevent blockade of neural data by stimulus artifact, CMOS switches (ADG714) are used to block the stimulation current from transmitting to the recording system. Each switch is connected between the electrode and the recording system and is synchronized with the stimulator to be triggered a short time before and after the stimulation pulse. During this time, the switches connecting the electrodes to the recording module (S2) will be kept open and the switches connecting the stimulator to the same electrodes (S1) will be closed (Figure 3a).

One challenge to using CMOS analog switches to block the stimulus from the recording system is they contain parasitic components that affect the AC performance of the device (Figure 3b). This means that part of the stimulus may couple from the source to the drain of S2 during stimulation. S2 acts to minimize charge coupling into the amplifier but since the amplifier is typically set to a gain of at least 60 dB, even small voltages may generate long contaminated signals.

To dissipate the charge coupled across the switch during stimulation, a 100 Ω resistor is used at the input of the amplifier during stimulation to suppress ringing. This resistor is also used to absorb any instantaneous charge coupled from the electrode to the amplifier when the electrode is reconnected to the amplifier. The resistor is disconnected from the amplifier when recording is resumed using another CMOS switch (S3) (Figure 3a). The timing of the switches depends on the shape of the voltage transient waveform across the electrode in response to a given stimulation pulse and is determined by the electrochemical properties of the interface as described in the section “Electrochemical Properties.”

### Electrochemical Properties

The shape of the voltage transient across a microelectrode is a factor of the electrochemical processes at the interface which can be estimated with an electrical equivalent circuit model. The model consists of an electrolyte resistance (*R*_s_) in series with the parallel combination of a double layer capacitance (*C*_dl_) and an impedance of faradaic reactions (*R*_P_) (Figure 3c; Conway, 1991). Equation 2 describes the relationship between an applied constant current pulse (I) and the resulting voltage (V) across the interface:

(2) $$V\,\left(t\right)=I\,R_{\text{s}}+I\,R_{\text{p}}\,\left(1-e^{-\frac{t}{R_{\text{P}}\,C_{\text{dl}}}}\right)$$

An example of the voltage transient in response to a stimulation current pulse of 60 μA, 200 μs is shown in Figure 3d.

In this model, capacitive charge injection represented by *C*_dl_ involves physical absorption and desorption of ions in an electrolyte. Faradaic reaction represented by *R*_P_ involves local donation of electrons through oxidation and reduction reactions, which is less desirable than a capacitive process since it involves formation of new species (Cogan, 2008). Both charge injection mechanisms may be involved during stimulation (irreversible faradaic reactions are to be avoided) (Merrill et al., 2005). Thus, the time constant of the electrode is governed by:

(3) $$\tau =R_{\text{p}}^{*}\,C_{\text{dl}}$$

**FIGURE 4:**
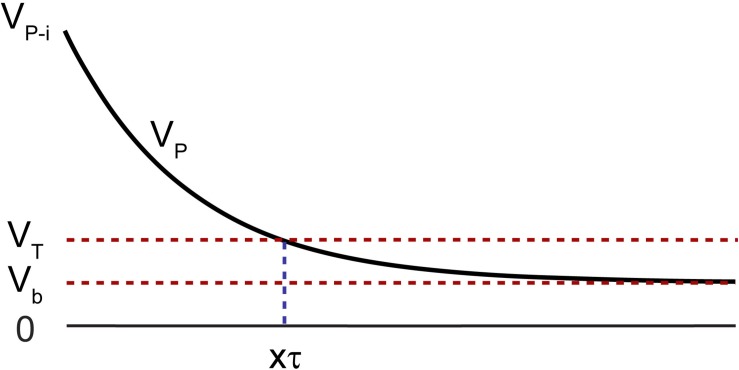
Polarization voltage (V_P_) across the electrode with respect to a reference of the same material after termination of the stimulation pulse with an initial value of V_P–i_. The voltage must drop below V_T_ before recording is resumed, to avoid amplifier saturation, which takes a time duration of multiple factors of τ represented as xτ.

After the termination of the stimulation pulse, the electrode is left with an initial polarization voltage (V_p–i_) and takes several τ’s to reach to a value close to its initial bias level (V_b_) (Figure 4). V_p–i_ is defined as:

(4) $$V_{\text{p}\,\_\,\text{i}}=V_{\text{b}}+\left(\Delta \,V-V\,a\right)$$

where V_a_ is the instantaneous voltage drop after the termination of the current pulse and ߡ V is the maximum voltage the electrode reaches at the end of the pulse (Bard and Faulkner, 2001). V_b_ is typically a few millivolts with respect to a large return electrode of the same material, as is the case of electrophysiology experiments. It is often difficult to identify V_a_ in a voltage transient plot, thus V_p_i_ cannot be accurately determined. Alternatively, V_P_max_, the maximum polarization voltage across an electrode to avoid potential exertion beyond the water window, may be used as V_p–i_. This is a worst-case scenario. This value is experimentally determined and is electrode material dependent (Cogan, 2008).

It is important that the polarization voltage on the working electrode (V_p_) with respect to a reference of the same material, drops to below a threshold before recording is resumed to avoid saturation of the amplifier. This threshold is governed by the settings on the recording amplifier such that:

(5) $$V_{\text{p}}<\frac{V_{\text{max}-\text{amp}}}{\text{Gain}}={\text{V}}_{\text{T}}$$

where V_max–amp_ is the maximum output voltage from the recording amplifier, and Gain is the gain of the amplifier.

**FIGURE 5:**
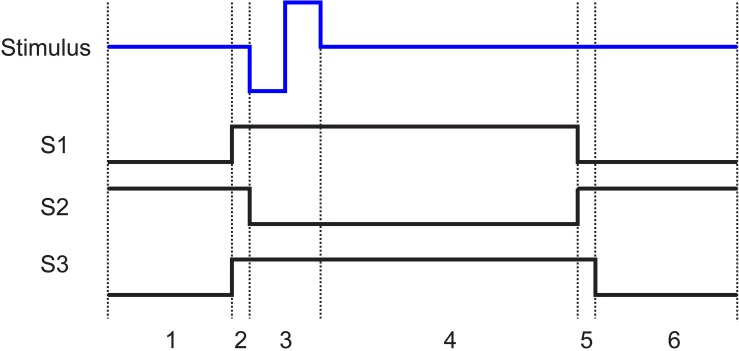
The state of the switches (1, closed; 0, open) shown in Figure 3a with respect to the stimulation pulse. In all cases when the stimulator is not pulsing, it is connected to ground. States 1 to 6 are as follows: 1. Recording: The electrode is connected to the recording amplifier while the stimulator and the resistor are disconnected. 2. Grounding: 200 μs before stimulation, the stimulator is connected to the electrode and the resistor is connected to the input of the amplifier. 3. Stimulation: The amplifier is disconnected from the electrode. The stimulation pulse is then applied to the electrode. 4. Discharge period: The electrode stays disconnected from the amplifier until the polarization voltage on the electrode falls below V_T_. 5. Discharge of residual charge (200 μs): The electrode is disconnected from the stimulator and connected to the recording system. The resistor remains connected to the input of the amplifier to absorb any charge injection due to residual offset on the electrode. 6. Recording resumed: The termination resistor is disconnected, and recording is resumed.

After the termination of the stimulation pulse, the electrode will take several time constants (xτ) to approach to below V_T_, which dictates the time duration the electrode must stay disconnected from the amplifier. The timing and state of the switches are shown in Figure 5. (1) The stimulator and the resistor at the input of the amplifier are disconnected while the electrode is connected to the amplifier. This is the recording phase. (2) 200 μs before the initiation of the stimulation pulse, S1 and S3 close. (3) S2 then opens to disconnect the recording system and the stimulation pulse is applied to the electrode. (4) The switches stay in that state for the duration of the pulse plus a predefined xτ. (5) S1 then disconnects the electrode from the stimulator and S2 reconnects the electrode to the amplifier while the input of the amplifier is still connected to ground through the 100 Ω resistor to absorb any instantaneous charge injected from the electrode. (6) 200 μs later, the resistor is then disconnected by S3 and recording is resumed.

It is important to note that the electrochemical properties of an electrode must be characterized for different media to determine different time constants of the electrode-electrolyte. Factors that affect the value of the time constant include electrode material, geometric and effective area of exposed region, and the electrolyte impedance. Thus, an electrode should be separately characterized for *in vivo* or *in vitro* experiments.

### System Architecture

**FIGURE 6:**
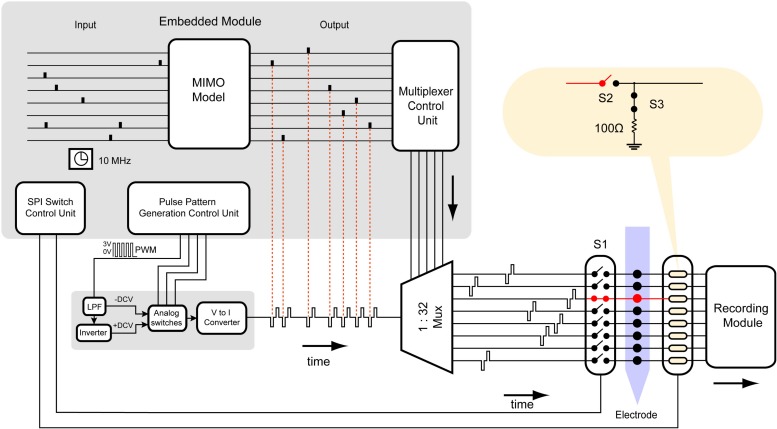
Block diagram and data stream of the multi-channel neurostimulator with SAS. The hardware is programmed by the embedded system to generate highly configurable constant current monopolar biphasic-pulse stimulation. Each block in the embedded system represents an algorithm to control the hardware. It consists of a MIMO non-linear dynamical model simulator, using a random number generator (RNG). The data is inputted to a multiplexer control unit to set the appropriate select lines of the multiplexer. The multiplexer control unit is synchronized with the SPI switch control unit to control CMOS switches.

Figure 6 illustrates the block diagram and data stream of the neurostimulator with the SAS technique. The microcontroller runs at a clock frequency of 10 MHz and is programmed with 4 blocks of control units: MIMO model simulator, pulse pattern generation control unit, multiplexer control unit, and switch control unit. The MIMO model simulator block simulates inputs from an external source such as the output of the MIMO model using a random number generator (RNG), which are generated by the microcontroller through recording noise from a floating general-purpose pin. It also uses another set of random numbers to vary the magnitude of the stimulus simulating feedback from neural response to stimulation. The timing and magnitude information across 32 channels are then collapsed into a single array. In case two channels need to be stimulated simultaneously (which is a rare event), one may be delayed by the duration of the biphasic pulse. The pulse pattern generation unit then translates the information into commands controlling the single channel neurostimulator to generate biphasic pulses with varying timing and magnitude. Next, the multiplexer control unit activates proper select lines of the multiplexer to send each stimulus to the target channel.

The switch control unit, which is synchronized with the MIMO model simulator, controls timing, and state of each switch used to suppress the stimulus artifact. The switches for each channel may be controlled individually, but because the stimulus artifact will contaminate all channels within the same media, the stimulation should be blocked from all recording channels.

### Power Consumption

The total power dissipation is dependent on the load current and the quiescent current. The output load current is dependent on the driving load (the electrode) and the stimulus waveform (pulse amplitude, pulse duration, and pulse rate), which is a variable defined by the user. Quiescent power consumption is the product of the current drawn by the supply (Icc) and the supply voltage (Vcc). Here, the quiescent power dissipation from all active parts except the microcontroller and multiplexer is 815 μW. The multiplexer consumes 60 μW. Thus, without multiplexing the system power consumption would be 815 μW^∗^32 = 26 mW, whereas multiplexing reduces this number to 875 μW. The microcontroller power consumption is dependent on usage of general-purpose input output pins. To generate arbitrary pulse patterns in real-time through a single channel, 4 pins are required by the pulse pattern generation control unit (Figure 6). Without multiplexing, the number of required pins would be 32^∗^4 = 128 for 32 channels. Whereas, with multiplexing, this number would be reduced to 9. Thus, multiplexing greatly reduces power consumption and real-estate usage.

### System Cost

The cost to fabricate and assemble the PCB is approximately $100. The minimum requirements are a personal computer, a TI MSP430 launchpad, and the Code Composer Studio to upload the code.

## Experimental Methods

### Design Characterization

The microcontroller was programmed to generate random numbers dictating both the timing and amplitude of pulses across each of the 32 channels independently. The range of amplitudes (I_max_) can be selected based on the user need and is limited to the supply voltage (V_supply_) and the total impedance of the electrode-tissue interface (|z| _electrode_)

(6) $$I_{\max}=\frac{V_{\text{supply}}}{{\left|z\right|}_{\text{electrode}}}$$

|z|_electrode_ includes the electrolyte resistance plus the polarization impedance across the electrode-electrolyte interface, which is frequency dependent. An electrochemical impedance spectroscopy (EIS) of the electrode is used to determine |z|_electrode_ at a frequency equal to the inverse of the pulse duration. This frequency is a reasonable approximation for non-sinusoidal pulses.

A Tungsten microelectrode was used to evaluate our system’s capability to minimize stimulus artifact. The microelectrode was first characterized by its electrochemical properties, namely the magnitude of each element in the equivalent circuit model shown in Figure 3c, its charge storage capacity and its τ using cyclic voltammetry (CV) and EIS performed by Gamry Reference 600 potentiostat (Gamry Instruments, Warminster, PA, United States). The return electrode was also made of Tungsten which was many times larger in area than the working electrode (WE).

**FIGURE 7:**
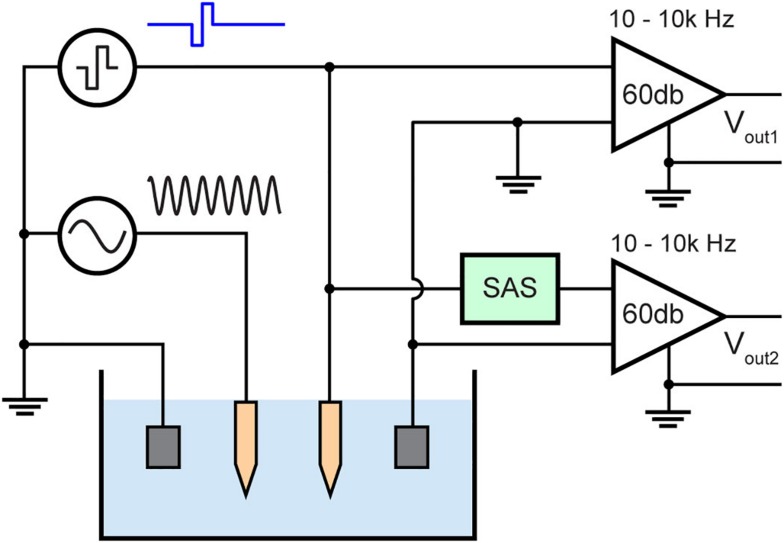
Test bench setup for evaluating the stimulus artifact with (V_out__2_) and without (V_out__1_) the SAS technique. The setup consists of diluted PBS mimicking neural tissue and a sinusoidal signal (1 kHz, 10 mV peak to peak) applied to a neighboring electrode mimicking neural activity.

Stimulation and recording experiments were performed in a phantom to compare the artifact with and without the SAS component. The phantom was 1/6 diluted PBS mimicking brain tissue impedance of approximately 0.25 S/m (Kandadai et al., 2012). To mimic neural activity, a known input of 1 kHz sinusoidal signal was applied to another microelectrode in the same solution. 1 kHz was chosen because it is within the spectral range of single unit activity. Also, this frequency over the frequency of evoked potentials provides better visualization for the earliest point at which biological signals may be recovered from the stimulus artifact. The amplitude of the input sinusoid is 10 mV peak to peak (Figure 7). The electric field from this electrode to the recording electrode would be attenuated due to distance and electrolyte impedance. This is also the case for *in vivo* recordings as the source of an action potential is in millivolts and distance and tissue impedance from the neuron to the recording electrode results in recordings of few hundred microvolts.

The time duration when no sinusoidal signal can be recorded due to the artifact is measured and compared. The recording amplifier used (A-M systems, model 1700) was set to 60 dB gain and a 10 Hz–10 kHz band pass filter. The maximum output voltage of this amplifier is 10 V, thus the voltage at the input of the amplifier must be less than 10 mV to avoid saturation. All signals were digitized and recorded by a recording system (Digidata 1322A, Molecular Devices) and data were saved by pClamps9 (Molecular Devices) software using a 100 kHz sampling frequency.

### *In vivo* Evaluation

**FIGURE 8:**
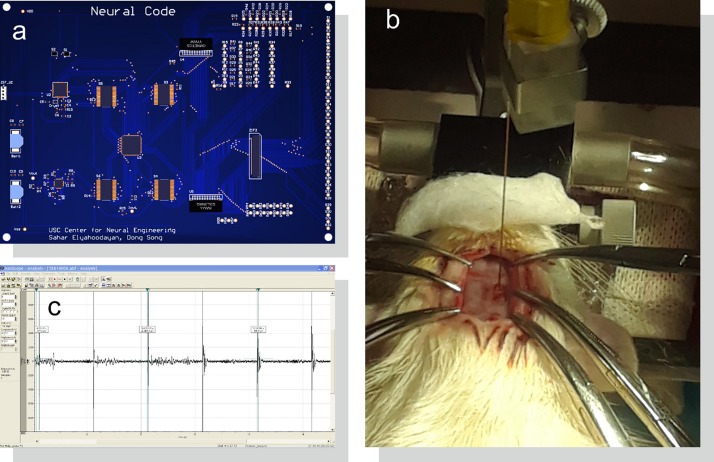
Acute *in vivo* setup for system evaluation. **(a)** PCB design of the system as shown in Figure 6 connected to **(b)** an implanted microelectrode in rat CA1 region of the hippocampus. **(c)** Stimulus artifacts and evoked neural responses are amplified, digitized, and saved with a bench-top neural recording system.

To demonstrate the system’s functionality *in vivo*, microelectrode recordings were conducted in dorsal hippocampus of one male Sprague-Dawley rat (12 weeks old, 350 g) using our designed and fabricated PCB (Figure 8a). All procedures were performed in accordance with protocols approved by the Institutional Animal Care and Use Committee of the University of Southern California. The anesthetic induction was carried out in a vaporizer-controlled induction chamber with a mixture of 4% isoflurane and O_2_. The rat was then anesthetized with a mixture of Ketamine and Xylazine. Once the animal was deeply anesthetized, it was placed on the surgery table. During the surgery, anesthesia was maintained with an inhalation of isoflurane (1∼2% in pure oxygen) administered with a nose cone from isoflurane machine with a scavenging cartridge attached. The status of anesthesia was checked periodically (every 15 min) by pinching the toe of the hind paw. If a positive “toe pinch” response was elicited, the doses of gaseous anesthesia would be increased. In addition, before, during and after the surgical procedure, the respiratory rate, mucous membrane color, and body temperature of the rat were monitored.

Ear bars on a stereotaxic frame were used to hold the rat’s head in place. Craniotomy of 2 mm × 4 mm was made over the right dorsal hippocampus and the dura was incised. The electrode was inserted at 2.80 mm posterior to the bregma and 2.50 mm lateral to the midline at a depth of 2.65 mm, perpendicular to the brain surface. A micro-manipulator was used to insert the electrode (Figure 8b). Two reference electrodes were inserted far away from the WE in the hindbrain; one for the stimulator and one for the recording system. Data was then recorded and saved by the recording system (Figure 8c).

Three sets of experiments were performed *in vivo*: (1) stimulate and record from the same electrode without the proposed SAS to measure the artifact, (2) stimulate and record from the same electrode with the SAS, and (3) repeat (1) and (2) after the animal is euthanized to separate neural responses from the stimulus artifacts.

## Results

We have successfully designed, fabricated and tested a multiplexed 32-channel microstimulator that can generate arbitrary spaciotemporal pattern of pulses driven by an external source and a SAS technique for recording from the same electrodes for feedback control of stimulation parameters. System characterization including examination of asynchronous arbitrary pulse pattern generation and evaluation of SAS in a phantom preparation are presented below. Finally, we present results of *in vivo* testing of this system on evoking and recording neural activities in the hippocampus.

### Asynchronous Arbitrary Pulse Pattern Generator

**FIGURE 9:**
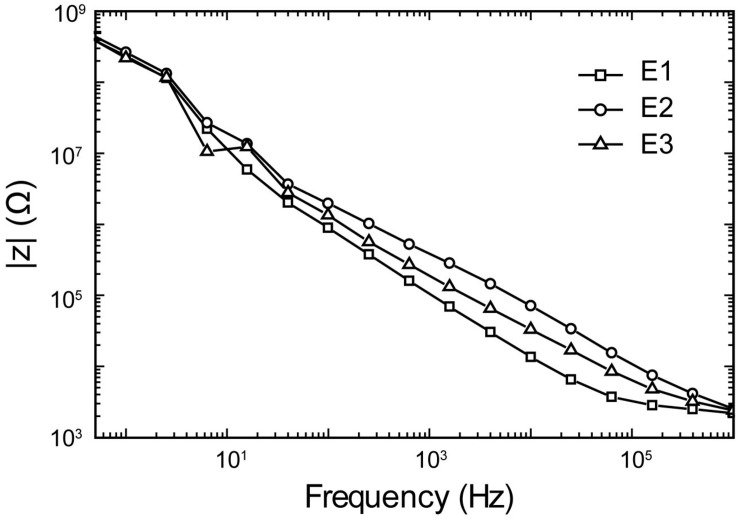
Representative electrochemical impedance spectroscopy plots of impedance magnitude in 1XPBS for three trials (E1–E3) of a Tungsten microelectrode used in all experiments.

The electrochemical impedance spectroscopy plot of the microelectrode is presented in Figure 9. A pulse duration of 200 μs (5 kHz) corresponds to a |z| _electrode_ of 50 kΩ. Thus, the stimuli were applied across a 50 kΩ resistors, mimicking |z| _electrode_, with amplitudes ranging from ±1 μA to ±60 μA, and the pulse intervals ranging from 0.5 to 100 ms. The system can be programmed to generate cathodic or anodic first stimulation pulses across individual channels. This choice is dependent on the region of the brain being stimulated as one waveform would manifest a lower threshold than the other.

**FIGURE 10:**
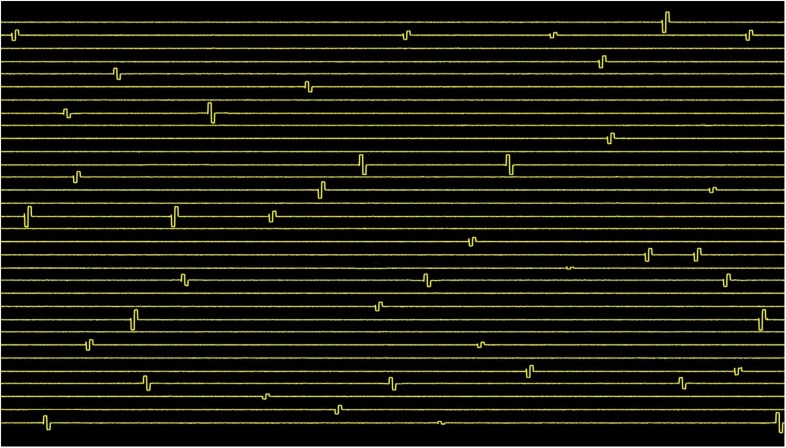
Spatiotemporal pattern of cathodic or anodic first stimulation pulses with varying amplitudes and intervals generated by the 32-channel neurostimulator. The stimuli were applied across 50 kΩ resistor mimicking |z| _electrode_. The amplitudes of the pulses range from ±1 μA to ±60 μA, and the pulse intervals range from 0.5 to 100 ms.

The result of the arbitrary stimulation pulse generator across 32 channels is shown in Figure 10 demonstrating the capability of the system to generate neural code-based stimulation with each channel generating either anodic first or cathodic first pulse. Small positive spikes at the beginning of the biphasic pulses are charge injection when switching from one channel of the mux to another. This is a value of maximum 5 pC, which is discharged during grounding phase.

The limitation of this system is imposed by the multiplexer. Multiplexing prevents two or more electrodes from being stimulated simultaneously. Instead, one stimulation pulse needs to be delayed by the duration of the pulse. A typical pulse duration to evoke neural response is 100–200 μs in small animals such as rats (Hambrecht, 1995; McCreery et al., 1998). If the pulse duration per phase is set to be 200 μs and two electrodes need to stimulate simultaneously, one pulse would be delayed by 400 μs. This is in fact rarely needed due to the sparse nature of neural spiking activities. Furthermore, our design specifications were based on hippocampal memory prosthesis application, which uses weak stimulation pulses to activate small and localized population of neurons. However, synchronized stimulation has also been shown effective in current steering and focusing (Chaturvedi et al., 2012; McIntyre et al., 2015; Steigerwald et al., 2016). Our system is a 32 n channel neurostimulator, where n is the number of individual current sources. Therefore, the design may easily be expanded to larger channel counts with individual current sources to allow synchronous stimulation through multiple channels.

### Electrochemical Properties

Cyclic voltammetry and electrochemical impedance spectroscopy were performed to assess the electrochemical properties of the microelectrodes used in the experiments. The measurements were analyzed with Echem Analyst (potentiostats and electrochemical instrument software by Gamry Instruments) to generate the values listed in Table 1. The cathodic charge storage capacitance of the electrode measured at a scan rate of 100 mV/s is 20 nC. The maximum charge applied to this electrode was 12 nC (60 μA^∗^200 μs). This is below the cathodic charge storage capacity of the electrode, which is an approximation of the charge injection capacity (Cogan, 2008).

**TABLE 1:** Electrochemical parameters of the microelectrode used in the experiments.

***C*_dl_**	***R*_P_**	***R*_S_**	**Charge storage capacity**	**Time constant (τ)**
0.3 nF	1 MΩ	10 KΩ	20 nC	300 μs

The V_p_ on the electrode after termination of the maximum stimulation pulse of 60 μA, 200 μs is 0.5 V (Figure 3d). After 5τ, V_p_ reaches to 0.5e^–5^ = 3.3 mV with respect to a large Tungsten return electrode. 5τ is chosen because the voltage across the electrode must reach to a value less than the input range of the amplifier to avoid saturation, which here is 10 mV. The τ of this electrode is 300 μs (*C*_dl_^∗^*R*p), and 5τ corresponds to 1.5 ms. Thus, a duration of 1.5 ms was spent after the termination of the stimulation before the electrode is reconnected to the amplifier.

### Evaluation of Stimulus Artifact Suppression

**FIGURE 11:**
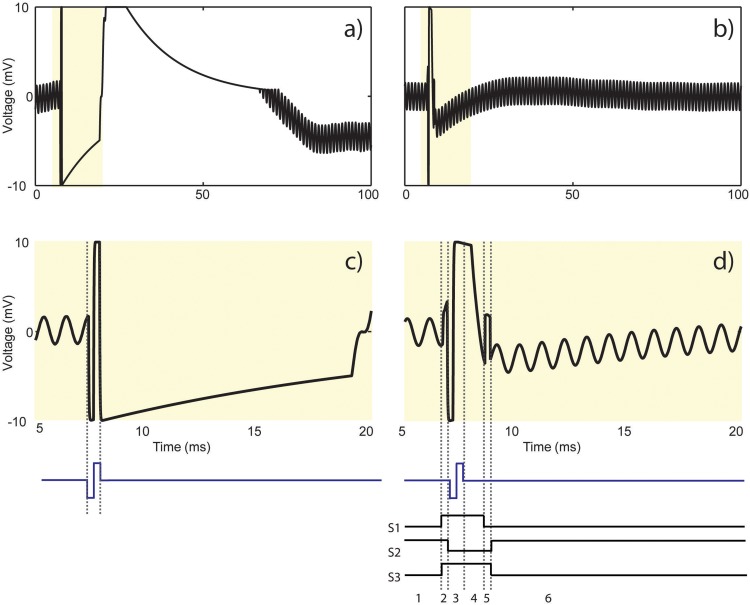
Results from the test bench experiment shown in Figure 7. Stimulus artifact **(a)** without and **(b)** with the SAS in response to a current pulse (cathodic first, 60 μA, 200 μs). Recording is resumed more than **(a)** 60 ms as opposed to **(b)** 2.3 ms after the onset of stimulation. **(c)** 15 ms time window from **(a)** demonstrating amplifier saturation and signal reflection. **(d)** 15 ms time window from **(b)** demonstrating the results of states 1 to 6 described in Figure 5 and summarized as follows: 1. Recording phase, 2. switching artifact (200 μs), 3. charge coupling across S2 during stimulation (400 μs), 4. discharge period of the electrode (1.5 ms), 5. switching artifact plus residual charge across the electrode (200 μs), and 6. resume recording.

Figure 11 demonstrates the resulting stimulus artifact in a phantom preparation with and without the SAS component. The 10 mV sinusoidal input signal is attenuated and recorded at less than 1 mV by the recording electrode. The input signal takes >60 ms to recover from the stimulus artifact when the electrode is directly connected to the recording amplifier (Figure 11a). By contrast, when the same signal is applied with the SAS component between the electrode and the amplifier, the recovery period is reduced to 2.3 ms (Figure 11b). Based on the SNR of the recorded signal, lower amplitudes may also be detected. A 15 ms time window of the two signals is shown in Figures 11c,d with respect to the timing of the stimulation pulse for better visualization.

Figure 11c shows that the stimulation pulse saturates the recording amplifier, causes ringing and finally settles according to the time constant of the electrode and the recording system. Figure 11d illustrates the resulting signal with respect to the 6 states of S1–S3 shown in Figure 6. State 1 is the recording phase. The artifact seen in state 2 is due to switching artifact caused by charge injection during closing of S1 and S2. State 3 shows the extra charge coupled from the source to the drain of S2 during stimulation, which still saturates the amplifier. The extra charge coupled is absorbed by the resistor connected at the input of the amplifier. The discharge period of the electrode is represented in state 4. The artifact in state 5 is due to switching artifact and possibly any residual charge left on the electrode when the electrode is reconnected to the amplifier. In state 6, recording is resumed but the sinusoid takes some time to recover to the baseline.

The control to compare recordings without the SAS technique is dependent on the amplifier and its settings. For example, a lower gain and higher high pass cut-off frequency will result in faster recovery period. Another factor affecting the recovery period is the input impedance of the amplifier. A lower input impedance would mean that the charge from stimulation would be divided between the electrode and the amplifier, which is undesirable. However, the settling time will be shorter and vice versa. The SAS technique is designed to be used with a variety of amplifiers. It minimizes charge coupling between the stimulator and the recording system and suppresses ringing due to amplifier saturation while using a wide-band filter.

It is apparent that our system is not capable of stimulation and recording perfectly simultaneously. However, it is important to note that neural tissue does not instantaneously respond to the stimulation pulse. Instead, it requires a minimum amount of time termed latency to generate the evoked response. The duration of latency depends on the properties of the target excitable tissue (Irnich, 1980).

### Acute *in vivo* Animal Validation

We stimulated the CA1 region of the hippocampus in anesthetized rats using increasing stimuli ranging in amplitude from 10 to 60 μA in increments of 10 μA separated in time by 1 s. Neural response was recorded with and without the SAS component. Each trial was repeated 3 times with a 5-minute recovery period between trials.

**FIGURE 12:**
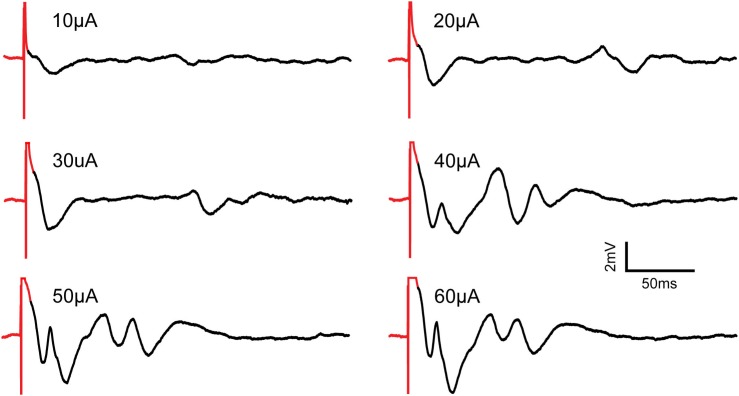
Recording of evoked neural responses to increasing micro-stimulation amplitudes in the CA1 region of the hippocampus of an anesthetized rat using the SAS component. All pulses are constant-current, cathodic-first and 200 μs in duration. Stimulus artifacts are shown in red. Evoked potentials are shown in black. As stimulation amplitudes increase, the evoked potentials show increased amplitudes and more complex waveforms.

Figure 12 shows neural responses following stimulation using the SAS component. Evoked potentials are apparent at stimulus amplitudes of 10, 20, and 30 μA. The amplitudes and durations of the evoked potentials increase with the stimulus amplitude. At and above 40 μA, complex waveforms with increased magnitudes are observed, possibly because more neurons are recruited, and more complex neural dynamic is elicited. The complex waveforms are characteristics of population spikes in the hippocampus as there is an initial depolarization of the nearby tissue followed by hyperpolarization deflections (Joëls and Fernhout, 1993).

**FIGURE 13:**
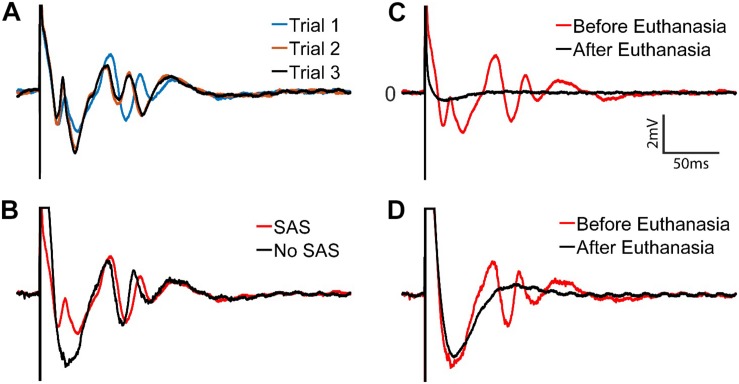
Stimulus artifacts and evoked potentials to a stimulation with 40 μA amplitude. **(A)** Three trials of recordings with the SAS. **(B)** Recordings with (red) and without (black) the SAS are overlaid for comparison. **(C,D)** Verification of the evoked potentials by comparing the signals before (red) and after (black) the rat was euthanized. Neural signals are retrieved within **(C)** ∼2 ms after the stimulation onset when SAS is used, and **(D)** ∼60 ms when SAS is not used.

Figure 13A shows results with the SAS component. Responses from 3 trials at 40 μA stimulus are overlaid to demonstrate the repeatability and variations of the responses. Comparison of the recorded signals with and without the SAS component is demonstrated in Figure 13B. Notably, short latency neural response is obscured when the SAS component is not used. To verify the recorded signals were indeed from neural tissue and clearly distinguish the artifact from neural activity, the experiment was repeated after the animal was euthanized as a control. Overlaying the signals recorded before and after euthanasia demonstrate clearly the effects of artifacts on the stimulating electrode with and without usage of SAS (Figures 13C,D).

**FIGURE 14:**
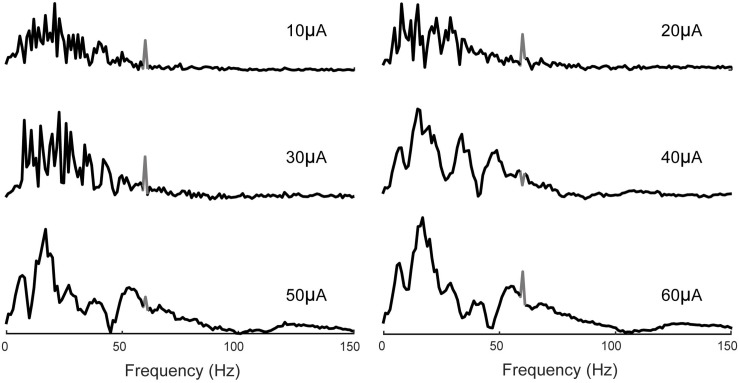
Frequency analysis of evoked potentials. 7–30 Hz band is apparent in all cases which increases in magnitude with the stimulus amplitude. Evoked potentials to higher stimulus amplitudes (40, 50, and 60 μA) also contain frequency components in the 40–100 Hz band. 60 Hz noise is shown in gray.

The frequency spectrum of the evoked potentials to stimuli of 10, 20, and 30 μA contains frequency components between 7 and 30 Hz. Furthermore, at higher stimulus amplitudes of 40, 50, and 60 μA, the neural responses also contain higher frequency components in the range of 40–100 Hz associated with Gamma oscillations previously studied in the CA1 region of the hippocampus (Bragin et al., 1995; Lega et al., 2016) (Figure 14).

## Discussion

We have designed, fabricated, and tested a versatile and cost-efficient neurostimulator that can deliver precise spatiotemporal patterns of stimulation pulses with arbitrary magnitudes and intervals through 32 channels continuously and in real time. This stimulator can be controlled by an external source such as a MIMO non-linear dynamical model to achieve localized and patterned micro-stimulation to the brain. The design also consists of a SAS component that allows stimulation and recording from the same electrodes with a very short delay. We have systematically tested this stimulator in a phantom preparation and then in anesthetized animals. Neural responses evoked by micro-stimulations are characterized and compared with and without the SAS technique to show its efficacy.

Highly configurable neuro-stimulators, and the ability to recording neural responses after stimulation is critical for implementing closed-loop neuromodulation or hippocampal memory prosthesis where consistent neural responses are desirable but often difficult to maintain. For example, variations in neural response may occur weeks or months after implantation due to inflammation. Inflammation causes glial cell encapsulation around the electrodes and thus weakens the neuron-electrode interaction, e.g., reduction of the stimulation effect and recorded signals (Polikov et al., 2005). Furthermore, neural plasticity may also contribute to variations of neural responses as the underlying neural circuits are constantly altered by behaviors (Kerr et al., 2011; Månsson et al., 2016). These variations can be compensated by adjusting stimulation parameters based on the feedback signals provided by the recording electrodes. Our system enables future studies to explore this possibility.

Our neurostimulator is particularly suitable for building hippocampal memory prosthesis. In a hippocampal memory prosthesis system, spatiotemporal patterns of stimulation to a downstream brain region are calculated based on the ongoing spatiotemporal patterns of neural activities in an upstream brain region using a predictive MIMO non-linear dynamical model. The stimulation patterns mimic the endogenous neural signals, which intrinsically are sparse, asynchronous, and involve multiple channels. The neurostimulator provides a way for delivering such patterns when connected to the output of a computational unit that contains the MIMO model.

A key feature of this design is the use of a multiplexer to save power and real estate to handle large numbers of electrodes. Higher channel counts are achievable with simple hardware and software modifications, with a complexity that scales sub-linearly with the channel counts. Future work includes a study to determine an optimum input-output ratio of the multiplexer for a hippocampal memory prosthesis. Another feature of the design is that it uses off-the-shelf microchips. As such, compared with commercially available neurostimulators such as Tucker Davis Technologies, Ripple Neuro, and Black Microsystems our system is a low-cost and reproducible design for use as a neuroscience tool.

For example, the Nano+Stim head stage by Ripple Neuro is a 32-channel stimulation and recording system with capability of generating complex pre-determined stimulation patterns and allowing rapid recovery following large stimuli from the same electrode^1^. However, this system utilizes a proprietary ASIC design that is not open to the public. The cost is approximately $9,500 per device for acute anesthetized experiments. By contrast, our system provides a design that costs approximately $100 per device, which can be easily scaled up to 32n channels, where n is the number of pulse generators.

Other systems described in the literature such as the ones developed by Lo et al. (2017) is a fully integrated closed-loop wireless neuromodulation system with 40 individual current sources for spinal cord stimulation. However, it does not include the function of generating arbitrary pulse patterns independently for each channel. The system developed by O’Leary et al. (2018) is a bidirectional arbitrary single channel waveform generator for adaptive seizure control, which suffers from low channel count. Tran et al. (2014) offers a CMOS 256 channel neurostimulator for retinal prosthesis, which is expensive and inefficient for a hippocampal memory prosthesis.

Stimulation and recording from the same electrode is another key feature required by a hippocampal memory prosthesis. The challenge in realizing this feature is imposed by direct connection of the stimulator and amplifier through a recording electrode. If a stimulation pulse causes amplifier saturation, the input signal would be clipped at the amplifier’s maximum input range. Consequently, neural response would be completely masked with this artifact and cannot be recovered. On the other hand, if recording is from a neighboring electrode to the stimulation electrode, or if the recording is from the stimulation electrode but the applied stimulus magnitude is small enough, the amplifier may not get saturated. In this case, often back-end signal processing may be used to separate artifact from neural response (Zhou et al., 2018).

Thus, a fundamental limitation of back-end signal processing approach is that it relies on unsaturated recordings of neural signals and stimulus artifacts, which are often unavailable due to the commonly encountered saturation of recording amplifiers. Even back-end signal processing of unsaturated recordings such as the ones proposed by Wagenaar and Potter (2002) and Wichmann (2000) face difficulties in separating neural activity from stimulus artifact due to their overlap in both time and frequency domains. More recently, Limnuson et al. (2015) developed a more sophisticated real time artifact suppression technique based on template subtraction, which requires a VLSI chip.

To avoid amplifier saturation, front-end artifact reduction has been implemented, which typically involves increasing the dynamic range of the amplifier to withstand larger voltages. This approach sacrifices power efficiency by using a higher voltage supply (Rolston et al., 2009) and still requires back-end signal processing to reduce the stimulus artifact. Another front-end approach is to subtract the artifact at the negative input of the amplifier based on a model that replicates the electrode-tissue properties (Nag et al., 2015). This technique holds promise but involves relatively complex computation and is to be validated in biological preparations.

Blanking techniques similar to our system have previously been used to reduce the artifact recorded from non-stimulation electrodes, where residual charge left on the electrode after termination of the stimulation pulse does not need to be accounted for Venkatraman et al. (2009), Cheng et al. (2017). The system that accounts for the discharge period of the stimulation electrode for same electrode stimulating and recording is by DeMichele and Troyk (2003). This system does not demonstrate data from bench-top system or neural tissue. Hottowy et al. (2012) designed a system with capability to generate spatio-temporal pattern of stimulation and stimulus artifact reduction. The range of stimuli used in this system to test the artifact rejection technique *in vitro* are low (0.43 μA, 100 μs), which did not cause amplifier saturation.

Our system is capable of recording and stimulating from the same electrode when a large stimulus of 60 μA, 200 μs are applied to the electrode. A large stimulus saturates the amplifier and causes ringing for tens to hundreds of milliseconds depending on the time constant of the electrode and the input impedance of the amplifier. To minimize charge coupling between the stimulator and the amplifier, prevent ringing, and allow discharge of the stimulation electrode, we describe the electrochemical characterization of the electrode to determine the timing of the 3 switches illustrated in Figure 5.

Lastly, state of the art neurostimulation integrated circuit has been realized in CMOS by Intan Technologies and utilized by many research groups (Ewing et al., 2013; Sessolo et al., 2013). The RHS2000 is a 16-channel stimulator/amplifier chip, which employs a fast recovery settling time scheme to minimize artifacts from stimulation. The technique Intan chip uses is “Low-frequency Cutoff shifting” to reduce recovery time from stimulation. This means that the user can switch the cut-off frequency of the amplifier high pass filter to a higher value during stimulation, effectively reducing the time constant and therefore the recovery period. However, this technique is mainly for stimulation and recording from neighboring electrodes. If it is implemented for same electrode stimulation and recording, the potential problem would be that some of the charge from the amplifier may couple into the amplifier and damage it over time. Plus, the amount of intended charge delivery to the electrode would be different than calculated.

Overall, to the best of our knowledge, no system similar to ours in terms of cost, scalability of stimulation channel numbers, capability of generating arbitrary stimulation patterns, and ability to stimulate and record from the same electrodes using large stimuli, has been tested and reported. Our immediate future work includes using smaller electrodes to record evoked response, as well as, spike trains. To make the electrodes suitable for stimulation and recording spike activities, we will use Pt-Ir electroplated electrodes which have shown to increase charge storage capacity without increasing the geometric area of the electrode (Petrossians et al., 2016; Cassar et al., 2019). We also aim to make the system fully closed-loop by real-time analysis of neural response to stimulation for use as input to our system for online adjustment of stimulation magnitude. We expect the final device to be a valuable tool for studying neurobiological basis of cognitive functions and a critical component for building cortical prostheses for restoring and enhancing cognitive functions.

## Data Availability Statement

All datasets generated for this study are included in the manuscript.

## Ethics Statement

This study was carried out in accordance with the recommendations of Institutional Animal Care and Use Committee (IACUC). The protocol was approved by the Department of Animal Resources of the University of Southern California (DAR, USC).

## Author Contributions

SE and DS contributed in design and bench-top characterization. WJ and HX performed *in vivo* implantation. SE, WJ, and HX performed *in vivo* recording and evaluation. SE wrote the first draft of the manuscript. All authors contributed to manuscript revision, read, and approved the submitted version.

## Conflict of Interest

The authors declare that the research was conducted in the absence of any commercial or financial relationships that could be construed as a potential conflict of interest.

## Footnotes

1 https://rippleneuro.com
